# Assessment of Aspiration Risk Using the Mann Assessment of Swallowing Ability in Brain-Injured Patients With Cognitive Impairment

**DOI:** 10.3389/fneur.2019.01264

**Published:** 2019-12-03

**Authors:** Shinyoung Kwon, Jaehoon Sim, Joonhyun Park, Youngsoo Jung, Kye Hee Cho, Kyunghoon Min, MinYoung Kim, Jong Moon Kim, Sang Hee Im

**Affiliations:** ^1^Department of Rehabilitation Medicine, CHA Bundang Medical Center, CHA University, Seongnam, South Korea; ^2^Rehabilitation and Regeneration Research Center, CHA University, Seongnam, South Korea; ^3^Department of Rehabilitation Medicine, CHA Gumi Medical Center, CHA University, Gumi, South Korea; ^4^Department and Research Institute of Rehabilitation Medicine, Severance Hospital, Yonsei University College of Medicine, Seoul, South Korea

**Keywords:** dysphagia, brain injury, cognitive impairment, dysphagia screening, videofluoroscopic swallowing study, Mann Assessment of Swallowing Ability

## Abstract

**Objectives:** The purposes of this study are to determine whether there is a correlation between the Mann Assessment of Swallowing Ability (MASA) and modified MASA (mMASA) according to various cognitive status and to investigate whether the cognitive status of patients with brain damage affects the prediction of aspiration using the MASA.

**Methods:** We retrospectively assessed 146 dysphagic patients with brain lesion due to various causes. Dysphagia was assessed using the MASA and mMASA. According to the videofluoroscopic swallowing study results, patients were divided into two groups: aspirators and non-aspirators. Patients were classified into four groups according to cognitive function according to the Korean version of Mini-mental State Examination scores: normal (>24), mild (21–24), moderate (10–20), and severe (<10) cognitive impairment. The correlation between the MASA and mMASA scores according to cognitive function were analyzed. The sensitivity, specificity, and positive and negative predictive values of the MASA scores for predicting aspiration were assessed.

**Results:** The MASA and mMASA scores showed a significant positive correlation in all cognition groups. In patients with more severe cognitive impairment MASA scores had high sensitivity and low specificity for prediction of aspiration. On the other hand, the MASA scores had low sensitivity and high specificity for prediction of aspiration in the normal and mild cognitive impairment groups.

**Conclusions:** The MASA and mMASA scores correlated with each other in patients with various levels of cognitive function. Interestingly, this study results demonstrated that patients with good cognitive function may have false negative results of MASA screening due to low sensitivity. Thus, when interpreting the MASA results, the impact of cognitive status should be taken into consideration.

## Introduction

Dysphagia is a disorder of the swallowing pathway resulting in impairment of the safety, efficiency, or quality of eating and drinking (1). It is one of the common and important complications of brain-injured patients, and its prevalence ranges from 30 to 67% in patients with stroke, traumatic brain injury (TBI), and brain tumors (2–5). Previous studies have shown that patients with dysphagia account for up to 50% of patients with acute stroke (6). In a clinical swallow assessment at 6 months post-stroke, 11% of patients had dysphagia, whereas in the videofluoroscopic swallowing study (VFSS), 50% of the patients had dysphagia (7, 8). Dysphagia causes complications, such as dehydration, malnutrition, asphyxia, and aspiration pneumonia, which may delay medical recovery and extend hospital stay (9). Aspiration pneumonia, which occurs in about 50% of cases of post-stroke dysphagia, even increases the mortality rate up to 45% (10). Additionally, patients with TBI are 79 times more likely to die of aspiration pneumonia than the general population (11). As early screening of dysphagia and proper dietary control are emphasized for improving the rehabilitation outcome in brain-injured patients (11–13), screening tools are routinely used to assess the risk of dysphagia and aspiration.

The Mann Assessment of Swallowing Ability (MASA) was developed by Mann et al. in 2002 as a screening tool for identifying eating and swallowing disorders in patients with stroke. The MASA can be used to quantify the aspiration risk via a bedside test. When predicting dysphagia in patients with stroke, the sensitivity and specificity of the MASA are reported as 71 and 72%, respectively. Additionally, for prediction of aspiration, the sensitivity and specificity of the MASA are estimated as 93 and 55%, respectively (14). The MASA is now used for various diseases (14–16) and effective for predicting the incidence of aspiration in a mixed-disease population (17). In 2010, Antonios et al. statistically reviewed the original data of the MASA to identify critical items for developing a screening tool that is quicker and more accurate to use. As a result, a more simplified version of the MASA, the modified MASA (mMASA), was designed to utilize highly discriminant items. The mMASA showed a high sensitivity (92.0%) and specificity (86.3%) for a prediction of dysphagia (15).

Both the MASA and mMASA are sensitive and specific screening tools for dysphagia (18). However, it is unknown whether these tests are reliable regardless of patient's cognitive function. Patients with impaired cognitive function may not follow instructions and result in deviation of test results different from the actual condition. Because MASA and mMASA contain several items reflecting cognitive status, such as alertness, cooperation, and auditory comprehension (19), the achieved scores can be lower in patients with poor cognitive function.

Cognitive dysfunction is frequent in 11.6–56.3% of brain-injured patients (20). Those with lower cognitive function are more likely to have severe dysphagia (21). As the need for dysphagia with aspiration screening is high in patients with impaired cognitive function, it is necessary to investigate the effect of cognitive function on dysphagia screening test results. To our knowledge, no study has evaluated the impact of cognitive impairment on the MASA and mMASA scores. Therefore, the purposes of this study are to explore whether there is a correlation between the MASA and mMASA according to various levels of cognitive function and to investigate whether the cognitive function of patients with brain damage affects the prediction of aspiration using the MASA.

## Materials and Methods

This was a retrospective study of 146 patients who were referred to a university hospital for consecutive VFSSs between December 2015 and November 2017. Inclusion criteria were as follows: (1) a brain injury (stroke, traumatic brain injury, and brain tumor) confirmed by a neurologist using computed tomography or magnetic resonance imaging, (2) aged ≥18 years, (3) available dysphagia evaluation results from the VFSS, MASA, and mMASA those had been conducted within a week, and (4) available cognitive assessment results from the Korean version of the Mini-mental State Examination (K-MMSE), of which evaluation date did not differ with swallowing evaluations by more than a week. Exclusion criteria were as follows: (1) underlying neurological conditions other than brain lesions, such as spinal cord injury and degenerative diseases, (2) a history of diseases that can affect swallowing function, such as oropharyngeal disease and prior surgery of the neck or cervical area.

Through medical chart review, patients' sex, age, onset of brain damage, cause and area of brain lesions, and dietary status were collected. In addition, results of cognitive and swallowing function assessed by the K-MMSE, VFSS, MASA, and mMASA were collected.

This study was conducted in accordance with the recommendations of the institutional review board (IRB) of the CHA University hospital, and the protocol was approved by the IRB of the CHA University hospital. The informed consent was waived because of the retrospective study design (ethical approval number: 2017-04-056).

### Dysphagia Assessment

#### Mann Assessment of Swallowing Ability and Modified Mann Assessment of Swallowing Ability

The MASA consists of 24 clinical items comprising four main components: general patient examination (alertness, cooperation, auditory comprehension, aphasia, apraxia, and dysarthria); oral preparation phase (saliva, lip seal, tongue movement, tongue strength, tongue coordination, oral preparation, respiration, and respiratory rate for swallowing); oral phase (gag reflex, palatal movement, bolus clearance, and oral transit time); and pharyngeal phase (cough reflex, voluntary cough, voice, tracheostomy, pharyngeal phase, and pharyngeal response). The MASA score is measured using a 5-point to 10-point rating scale. The total score of the MASA is 200 points, and the cutoff value is 177 points. The results of the MASA are interpreted as no abnormality (≥178), mild dysphagia (168–177), moderate dysphagia (139–167), and severe dysphagia (≤138). The risk of aspiration are defined based on the total scores into four categories as follows: no abnormality (≥170), mild (149–169), moderate (141–148), and severe (≤140) (14). In this study, we identified the risk of aspiration based on only a MASA score of 170.

The mMASA consists of 12 items, and the total score is 100 points with a cutoff value of dysphagia as 94. It evaluates alertness, cooperation, respiration, expressive dysphasia, auditory comprehension, dysarthria, saliva, tongue movement, tongue strength, gag reflex, voluntary cough, and palatal movements. In addition, “no response” was added as a possible response in auditory comprehension (15). The MASA and mMASA are performed by a skillful occupational therapist with more than 15 years of experience in the evaluation of dysphagia. All of MASA, mMASA, and VFSS were conducted within a week.

#### Videofluoroscopic Swallowing Study

The VFSS protocol from Logemann's study (22) was performed by a physiatrist in the fluoroscopic radiography room. In a sitting position, the lateral views of the oropharynx and larynx were obtained while the patient was given consecutive boluses of semisolid, solid, thick liquid, and thin liquid barium preparations orally. Finally, the patient was asked to drink 30 ml of thin liquid. The test was stopped when aspiration was detected on the fluoroscopic image. The diagnostic criteria for aspiration are as follows (8): normal, no entry of contrast material through the true vocal cords; mild, a trace of contrast materials through the true vocal cords; moderate, entry of <10% of the bolus through the true vocal cords; severe, entry of >10% of the bolus through the true vocal cords; and complete, frank aspiration of materials through the vocal cords without an observable reaction by the patient. Fluoroscopic images were stored on the server system, and the physiatrist reevaluated the results based on the recorded images. According to the VFSS results, patients were divided into two groups: patients with aspiration (aspirators) and those without aspiration (non-aspirators). Patients with only mild penetration were classified as the non-aspiration group, and the severity of aspiration in aspirators was not determined.

### Assessment of Cognitive Function

Cognitive function was assessed by referring to the K-MMSE result noted in the patients' medical charts. The MMSE is a widely used evaluation tool, and it was initially designed for screening for dementia (23). In most studies, the criterion for the presence of cognitive impairment was indicated at 23–25 points, but the classification of severity varied (24–27). Thus, we classified the K-MMSE scores as follows: scores ≤24 were considered abnormal, 21–24 as mild, 10–20 as moderate, and <10 as severe cognitive impairment (25). Cognitive function evaluation and swallowing assessment were performed during a proximate period within 1 week.

### Statistical Analysis

All variables, except for age and K-MMSE, were found to be not normally distributed (Kolmogorov-Smirnov test) and did not meet parametric assumptions. Differences in the distribution of selected characteristics between the patient groups were examined using the chi-square test for categorical variables. Differences in continuous variables between the study groups were analyzed using either the Mann Whitney *U* test or *t*-test.

The correlation between the MASA and mMASA according to cognitive status was analyzed using the Spearman correlation coefficient. The data were analyzed using 2 × 2 tables to determine the sensitivity, specificity, positive predictive value, and negative predictive value of the MASA for predicting aspiration according to cognitive status. Sensitivity was calculated as the proportion of true positive aspiration detected during the VFSS. Specificity was calculated as the proportion of true negative tests in patients without aspiration. The predictive value indicated the probability of aspiration based on the test results.

The statistical analysis was performed using standard statistical software (SPSS version 21.1 for Windows, SPSS, Inc., Chicago, IL, USA). The significance level was set at 0.05.

## Results

**Figure 1:**
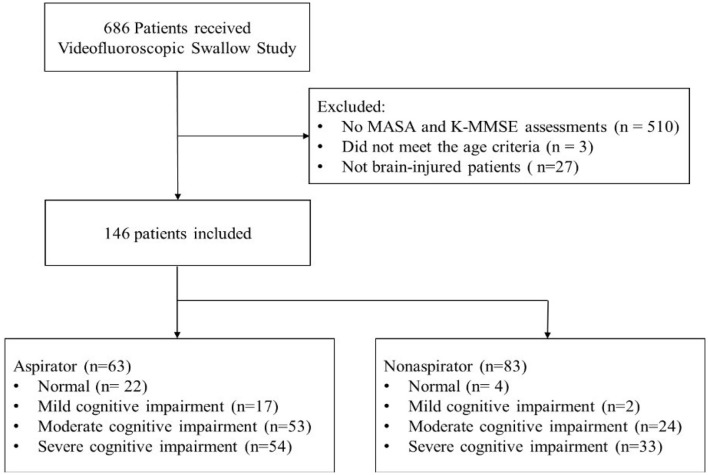
Flowchart of the study and analysis.

Among 686 patients, 510 patients were excluded because they had no records of the K-MMSE. Three patients did not meet the age criteria, and 27 patients were not brain-injured patients. Finally, 146 patients (90 men and 56 women; mean age 63.2 ± 15.9 years) with brain injury were included (51 with ischemic stroke, 58 with hemorrhagic stroke, 21 with TBI, and 16 with brain tumor) (Figure 1). According to the K-MMSE score, patients were divided into two cognitive function groups: normal (*n* = 22), abnormal (*n* = 124). The abnormal group was divided into three subgroups: mild impairment (*n* = 17), moderate impairment (*n* = 53), and severe impairment (*n* = 54). Table 1 summarizes the demographics and clinical characteristics of patients.

**Table 1:** Demographics and clinical characteristics of patients (*n* = 146).

**Variable**	**Total (*n* = 146)**	**Aspirator (*n* = 63)**	**Non-aspirator (*n* = 83)**	***P***
Age (years)	63.2 ± 15.9	61.5 ± 17.1	61.5 ± 17.1	0.216
Sex (*n*, M/F)	146, 90/56	63, 35/28	83, 55/28	0.187
Etiology (*n*, M/F)				0.632
Cerebral infarction	51, 24/27	19, 8/11	32, 16/16	
Cerebral hemorrhage	58, 41/17	28, 17/11	30, 24/6	
Traumatic brain injury	21, 17/4	10, 7/3	11, 10/1	
Brain tumor	16, 8/8	6, 3/3	10, 5/5	
Brain lesion side: Rt/Lt/bilateral	34/47/65	9/19/35	25/28/30	0.030^*^
Onset to evaluation: MASA (day)	67.0 ± 89.4	67.6 ± 61.6	66.5 ± 106.0	0.942
Onset to evaluation: VFSS (day)	66.8 ± 89.8	67.5 ± 61.9	66.3 ± 106.7	0.936
Interval between MASA and VFSS (day)	2.3 ± 2.0	2.2 ± 2.1	2.3 ± 2.0	0.660
K-MMSE (*n*, scores)	146, 13.5 ± 9.5	63, 9.6 ± 8.5	83, 16.5 ± 9.3	0.000^**^
Normal	22, 27.5 ± 1.3	4, 27.3 ± 0.5	18, 27.6 ± 1.5	0.690
Abnormal	124, 11.0 ± 8.1	59, 8.4 ± 7.3	65, 13.4 ± 8.1	0.000^**^

*Values are presented as mean ± standard deviation (SD)*.

* *p < 0.05*,

** *p < 0.01*.

The MASA and mMASA scores showed a significant positive correlation (*r* = 0.961, *P* < 0.01) (Table 2). In subgroup analysis of cognitive function, the MASA and mMASA scores also showed a significant correlation within all groups.

**Table 2:** Correlation between MASA and mMASA according to cognitive status.

**Cognitive status (*n*)**	**MASA**	**mMASA**	***r***
Total (146)	156.2 ± 43.1	80.3 ± 20.1	0.961^**^
Normal (22)	182.7 ± 22.4	93.7 ± 8.8	0.896^**^
Abnormal (124)	151.4 ± 48.3	78.0 ± 22.4	0.974^**^
Mild impairment (17)	184.2 ± 14.7	93.6 ± 7.3	0.845^**^
Moderate impairment (53)	169.0 ± 27.9	87.0 ± 11.1	0.926^**^
Severe impairment (54)	124.0 ± 47.9	64.2 ± 22.2	0.973^**^

*Values are presented as mean ± standard deviation (SD)*.

** *p < 0.01*.

The sensitivity and specificity of the MASA for predicting aspiration were 71.4 and 69.9%, respectively. In the subgroup analysis according to cognitive function, the subgroup with severe impairment revealed the highest sensitivity of 90.9 and the lowest specificity of 38.1. Normal cognition group had the lowest sensitivity of 25.0 and the highest specificity of 88.9. The patient group with more severe cognitive impairment tended to have higher sensitivity and positive predictive values, and lower specificity and negative predictive values of the MASA for prediction of aspiration. On the other hand, lower sensitivity and positive predictive values, and higher specificity and negative predictive values of the MASA for prediction of aspiration was observed in the normal and mild cognitive impairment groups (Table 3).

**Table 3:** Accuracy of MASA for prediction of aspiration according to cognitive status.

**MASA**	**Cognitive status (K-MMSE)**					
	**Total**	**Normal**	**Abnormal**	**Mild impairment**	**Moderate impairment**	**Severe impairment**
Sensitivity	71.4	25.0	74.6	50.0	50.0	90.9
Specificity	69.9	88.9	64.6	86.7	72.4	38.1
Positive predictive value	64.3	33.3	65.7	33.3	60.0	69.8
Negative predictive value	76.3	84.2	73.7	92.9	63.6	72.7

## Discussion

The results of this study shows the MASA and mMASA scores are significantly correlated regardless of patients' cognitive function. This finding suggests that the mMASA, which has a similar tendency with the MASA, might be used instead of the MASA for brain-injured patients with various degrees of cognitive impairment. Using the mMASA, dysphagia maybe readily and quickly screened in brain-injured patients. However, there is still a lack of research on the mMASA for a clinical application. Currently, the cutoff value of mMASA for dysphagia screening has been evaluated (15), without regards to the risk of aspiration. Therefore, the significance of mMASA in identifying aspiration risk needs to be studied further. As this study revealed, the sensitivity and specificity of the MASA for prediction of aspiration change according to patient's cognitive function. Accordingly, further study is needed to confirm the influence of cognitive function on the sensitivity and specificity of mMASA.

This study revealed the sensitivity and specificity of the MASA scores for predicting aspiration based on VFSS results. Previously, the sensitivity and specificity of the aspiration risk have been reported as 93 and 55%, respectively (14); however, the influence of cognitive function was not taken into consideration. In our study, the sensitivity and specificity were 71.4 and 69.9%, respectively. In the subgroup analysis, patient group with severe cognitive impairment revealed the highest sensitivity of 90.9 and normal cognition group showed the lowest sensitivity of 25.0. A high sensitivity is a prerequisite for a good screening test. In other words, the MASA is likely to be less considerable as a screening tool for brain-injured patients with good cognitive function.

The MASA includes the items of alertness, cooperation, and auditory comprehension related to patient's cognitive function as well as swallowing assessment items. As mentioned above, the MASA includes evaluation items for cognitive function (total scores of 30), and patients with cognitive impairment are more likely to have a low score and are classified as having dysphagia or a higher risk of aspiration. Therefore, in this study, the patients with low cognitive function were considered to have a high sensitivity and low specificity.

If a patient with good cognitive function has a good oral function and only a pharyngeal phase dysfunction, his MASA and mMASA scores can be high even the patient has aspiration. For this reason, the normal cognition group may not show significant difference in the MASA and mMASA scores between aspirators and non-aspirators. From this result, we draw a careful conclusion that the MASA may be of less value as a screening tool in patients with good cognitive function.

Cognitive impairment is a major and common problem in the patients with brain lesion. In a previous study, the relationship between dysphagia and cognitive impairment in brain-injured patients was introduced (19). Patients with attention, cognitive-perceptual, and behavioral disorders experience swallowing disorders (19, 28–30). Dysphagia is more clinically relevant in patients with non-amnestic mild cognitive impairment, as it may interfere with motor activities (31). Dysphagia after stroke is not only limited to brainstem involvement, and silent aspiration is more likely to occur (32). Patients with significant sensory deficits also have increased risk of aspiration (33). Brain-injured patients with severe cognitive impairment would have more severe swallowing problems and higher aspiration risk. However, prior studies have analyzed only the relationship between the MASA, mMASA and VFSS without consideration of cognitive function. To the best of our knowledge, this is the first study to assess the effect of cognitive function on the results of dysphagia screening tools.

This study defined the cognitive function level of the patients based on the MMSE. As this study utilized the retrospective data collection, the analysis of cognitive function had limited data set. The MMSE is a tool that was initially developed for diagnosing dementia (34), however, it is also widely used for evaluating cognitive impairment (25, 27). The MMSE has a weakness lacking assessment of frontal lobe function and focusing primarily on left hemispheric cognitive function (language, verbal memory, calculation, etc.) (27, 35). Nevertheless, the validity of the MMSE is still acceptable, and it has advantages of being easy to use and has a low inter-rater variability (27, 36–39).

The present study has other limitations. There is a lack of homogeneity in the subject population, including various etiology of brain damage. In addition, the number of subjects with good cognitive function was much smaller than those with severe cognitive impairment. Future studies should be conducted on more homogenous samples with various cognitive function to identify the cognitive effects on the results of dysphagia screening. Through the prospective studies, we should consider using cognitive assessment tools which represent the minor deficit and overall function of the brain (40, 41).

## Conclusions

The MASA and mMASA scores correlated with each other in patients with various cognitive function. However, the sensitivity and specificity of the MASA may vary depending on the cognitive level of brain-injured patients. Thus, the test results should be interpreted carefully considering the impact of cognitive function on the MASA scores. Especially for the patients with relatively good cognitive function, it is helpful to recognize the possibility of false negative result of MASA study for the prediction of aspiration.

## Ethics Statement

This study was conducted in accordance with the recommendations of the Institutional Review Board (IRB) of the CHA University Hospital, and the protocol was approved by the IRB of the CHA University Hospital. The informed consent was waived because of the retrospective study design (ethical approval number: 2017-04-056).

## Author Contributions

All authors listed have made a substantial, direct and intellectual contribution to the work, and approved it for publication.

### Conflict of Interest

The authors declare that the research was conducted in the absence of any commercial or financial relationships that could be construed as a potential conflict of interest.
